# Supplementary material for: Modeling the Impact of Vaccination on COVID-19 and Its Delta and Omicron Variants
Source: Viruses. 2022 Jul 6;14(7):1482. doi: 10.3390/v14071482 (PMC9319847; doi:10.3390/v14071482)
Supplement: Supplementary file 1 [file viruses-14-01482-s001.zip › viruses-1778116-supplementary.pdf]

Supplementary Materials for

Modeling the Impact of Vaccination on COVID-19 and its Delta Variant

Jianbo Wang[1,2], Yin-Chi Chan[1,3], Ruiwu Niu[3], Eric W. M. Wong[1,*], and Michaël Antonie van Wyk[1,5]

[1]Department of Electrical Engineering, City University of Hong Kong, Hong Kong, China
[2]School of Computer Science, Southwest Petroleum University, Chengdu, China
[3]Institute for Manufacturing, University of Cambridge, Cambridge, United Kingdom
[4]College of Mathematics and Statistics, Shenzhen University, Shenzhen 518060, China
[5]School of Electrical and Information Engineering, University of the Witwatersrand at Johannesburg, Johannesburg 2000, South Africa

Supplementary Figures

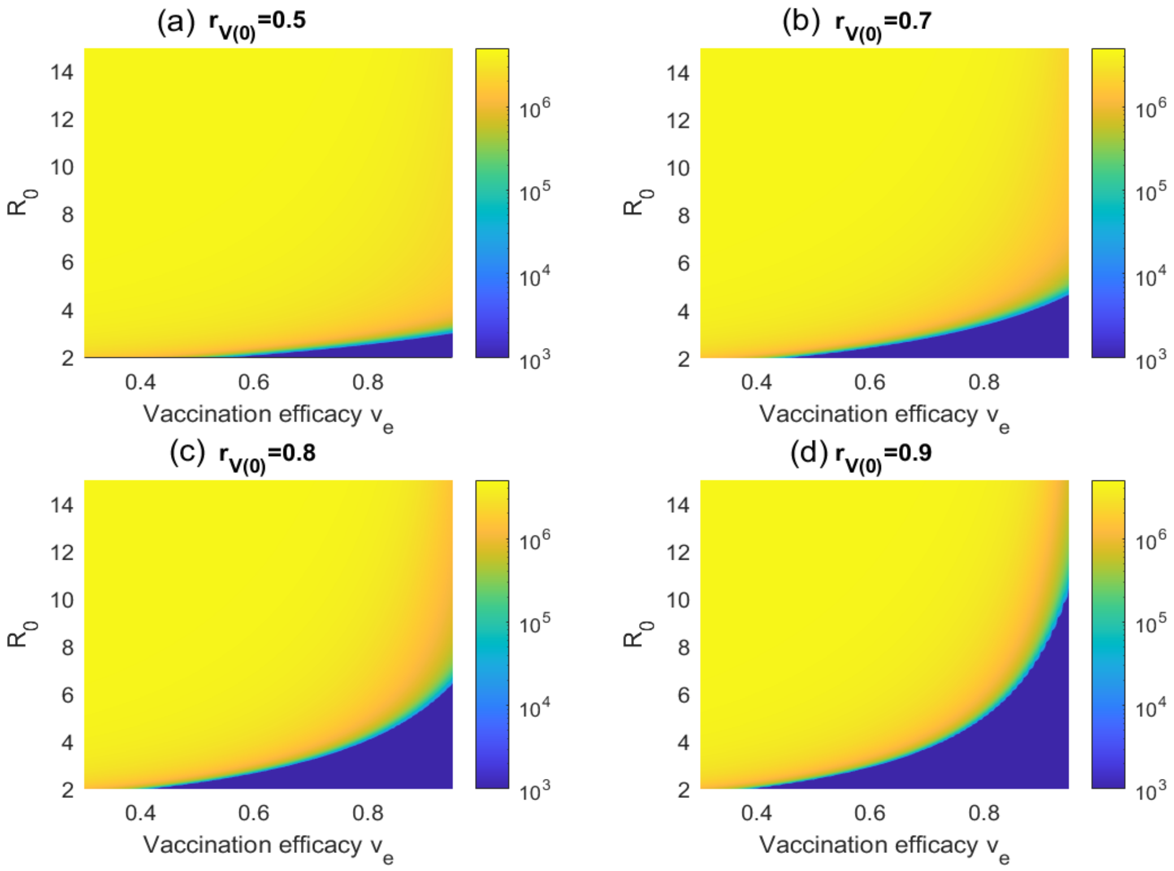

Figure S1: Cumulative hospitalizations $H_c(\infty)$ with respect to R_0 and the vaccine efficacy v_e, for a simulated Delta outbreak in Hong Kong and NPIs ($c = 0.2$).

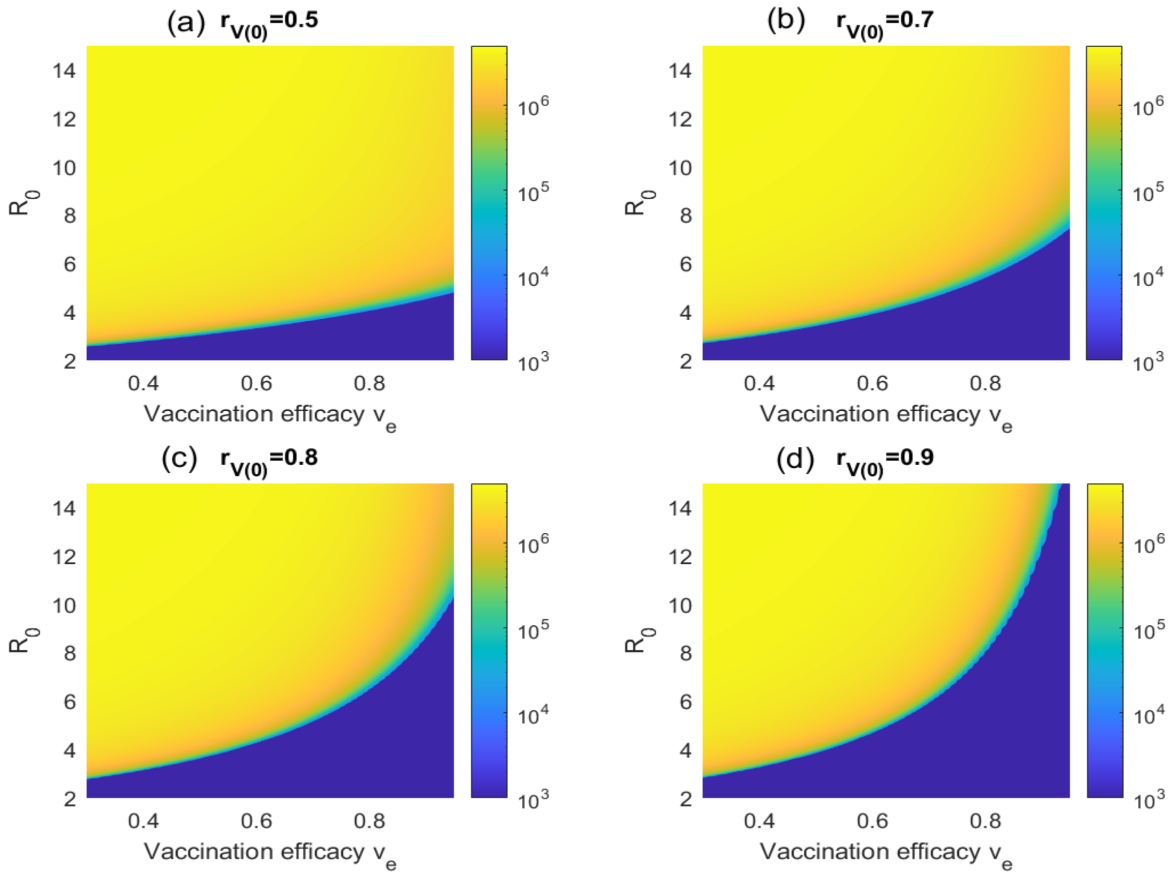

Figure S2: Cumulative hospitalizations $H_c(\infty)$ with respect to R_0 and the vaccine efficacy v_e, for a simulated Delta outbreak in Hong Kong and NPIs ($c = 0.5$).

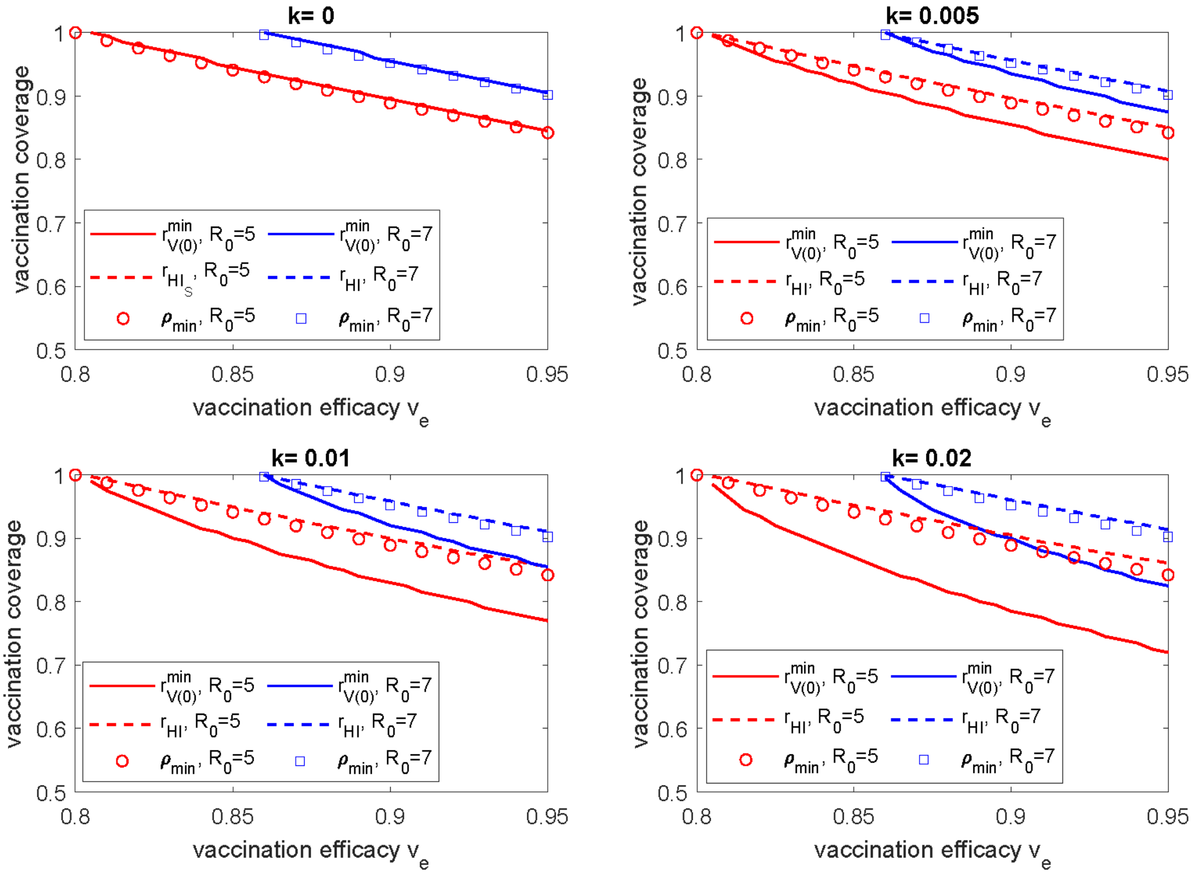

Figure S3: Minimum vaccination threshold required such that the cumulative number of hospitalizations is less than $10^{-4}N$, for different vaccine efficacies v_e and vaccination rates k, and no NPIs ($c = 0$). The theoretical herd immunity threshold $\rho_{\min}$ is also shown.

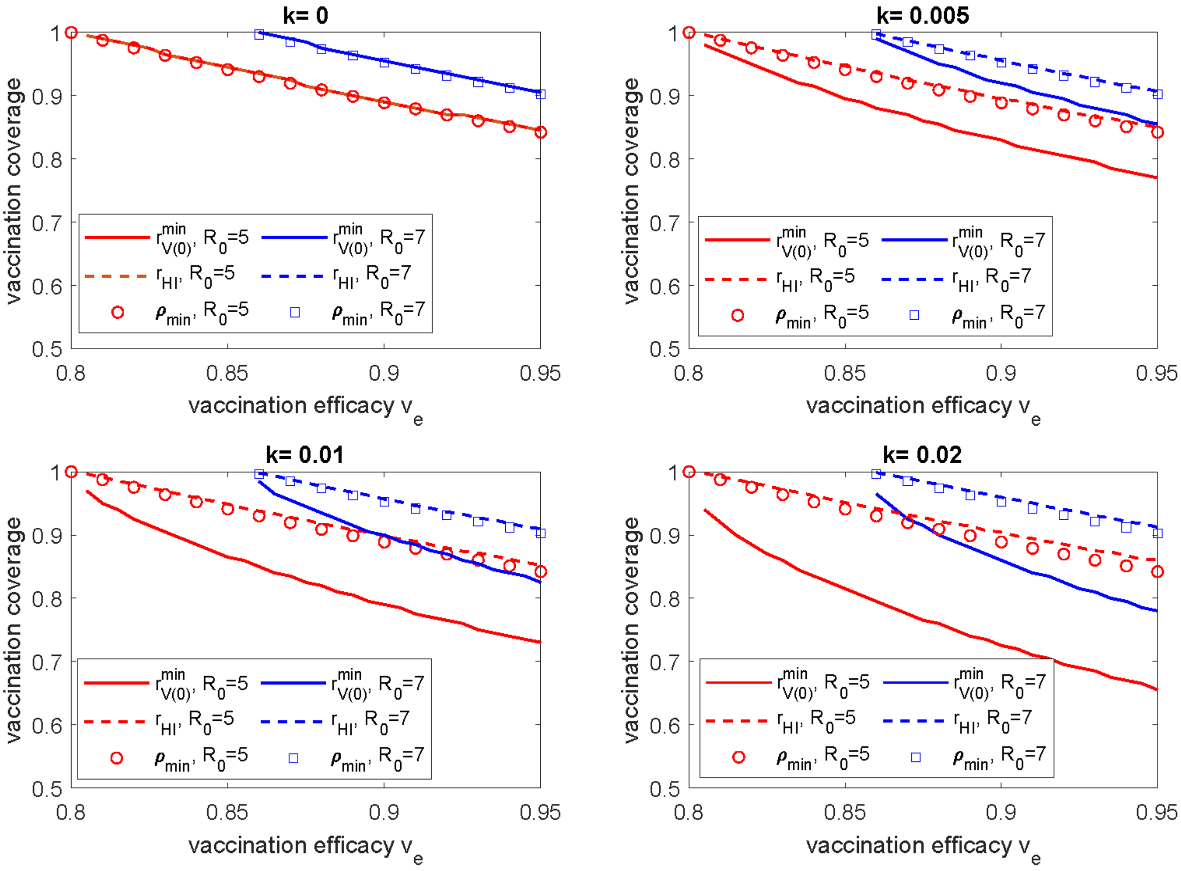

Figure S4: Minimum vaccination threshold required such that the cumulative number of hospitalizations is less than $10^{-3}N$, for different vaccine efficacies v_e and vaccination rates k, and no NPIs ($c = 0$). The theoretical herd immunity threshold $\rho_{\min}$ is also shown.
